# Genome‐wide insights into introgression and its consequences for genome‐wide heterozygosity in the *Mytilus* species complex across Europe

**DOI:** 10.1111/eva.12974

**Published:** 2020-04-24

**Authors:** David L. J. Vendrami, Michele De Noia, Luca Telesca, Eva‐Maria Brodte, Joseph I. Hoffman

**Affiliations:** ^1^ Department of Animal Behavior University of Bielefeld Bielefeld Germany; ^2^ Institute of Biodiversity, Animal Health & Comparative Medicine College of Medical Veterinary & Life Sciences University of Glasgow Glasgow UK; ^3^ Department of Earth Sciences University of Cambridge Cambridge UK; ^4^ British Antarctic Survey, High Cross Cambridge UK; ^5^ Alfred Wegener Institute Kurpromenade Germany

**Keywords:** genetic variation, genome‐wide heterozygosity, hybridization, introgression, *Mytilus*, Restriction site‐associated DNA sequencing (RAD sequencing), stock structure

## Abstract

The three mussel species comprising the *Mytilus* complex are widespread across Europe and readily hybridize when they occur in sympatry, resulting in a mosaic of populations with varying genomic backgrounds. Two of these species, *M. edulis* and *M. galloprovincialis*, are extensively cultivated across Europe, with annual production exceeding 230,000 tonnes. The third species, *M.* *trossulus*, is considered commercially damaging as hybridization with this species results in weaker shells and poor meat quality. We therefore used restriction site associated DNA sequencing to generate high‐resolution insights into the structure of the *Mytilus* complex across Europe and to resolve patterns of introgression. Inferred species distributions were concordant with the results of previous studies based on smaller numbers of genetic markers, with *M. edulis* and *M. galloprovincialis* predominating in northern and southern Europe respectively, while introgression between these species was most pronounced in northern France and the Shetland Islands. We also detected traces of *M. trossulus* ancestry in several northern European populations, especially around the Baltic and in northern Scotland. Finally, genome‐wide heterozygosity, whether quantified at the population or individual level, was lowest in *M. edulis*, intermediate in *M. galloprovincialis,* and highest in *M. trossulus*, while introgression was positively associated with heterozygosity in *M. edulis* but negatively associated with heterozygosity in *M. galloprovincialis*. Our study will help to inform mussel aquaculture by providing baseline information on the genomic backgrounds of different *Mytilus* populations across Europe and by elucidating the effects of introgression on genome‐wide heterozygosity, which is known to influence commercially important traits such as growth, viability, and fecundity in mussels.

## 1 INTRODUCTION

Mussel farming is one of the most important aquaculture sectors in Europe, with annual production exceeding 230,000 tons (FAO, 2015). The three mussel species present in Europe, collectively referred to as the *Mytilus* complex, readily hybridize when they occur in sympatry (Gosling, 1992). Two of them, *M. edulis* (hereafter referred to as *ME*) and *M. galloprovincialis* (hereafter referred to as *MG*), are extensively cultivated along the Atlantic coast of Europe as well as in the Mediterranean (Michalek, Ventura, & Sanders, 2016). By contrast, the third species, *M. trossulus* (hereafter referred to as *MT*) is undesirable for cultivation due to the possession of fragile shells and poor quality meat (Penney, Hart, & Templeman, 2007, 2008). Its hybridization with the other two species has been described as commercially damaging and has been linked to significant economic losses in regions of Scotland (Scott et al., 2010;Scottish Government, 2014).

Owing to the economic importance of mussels and because the *Mytilus* complex is ideally suited to exploring the processes that generate and maintain hybrid zones, several population genetic studies have sought to characterize the geographic distributions of these species across Europe and to identify areas in which introgression takes place. Studies using diverse genetic markers, from mitochondrial sequences through allozymes and microsatellites to single nucleotide polymorphisms (SNPs), have shown that *MG* is the dominant species in southern Europe (Daguin, Bonhomme, & Borsa, 2001;Sanjuan, Zapata, & Alvarez, 1994;Zbawicka, Drywa, Śmietanka, & Wenne, 2012), where it is found across the Mediterranean and along the coast of the Iberian Peninsula, whereas *ME* dominates the cooler northern European coastlines (Daguin et al., 2001;Zbawicka et al., 2012). By contrast, *MT* is better adapted to brackish conditions and occurs in the Baltic as well as in parts of Norway and Greenland (Kijewski, Zbawicka, Väinölä, & Wenne, 2006;Mathiesen et al., 2017;Stuckas et al., 2017;Väinölä & Strelkov, 2011;Wenne, Bach, Zbawicka, Strand, & McDonald, 2016;Zbawicka et al., 2012;Zbawicka, Sa´nko, Strand, & Wenne, 2014), while its hybrids have also been found in the Netherlands and Scotland (Beaumont, Hawkins, Doig, Davies, & Snow, 2008;Śmietanka, Zbawicka, Wołowicz, & Wenne, 2004;Zbawicka, Burzyński, Skibinski, & Wenne, 2010). Studies of hybridization between *ME* and *MG* have also revealed unforeseen complexities in France, the Netherlands, and the UK, where pure populations of the parental species coexist with mixed populations, resulting in complex species mosaics (Beaumont, Turner, Wood, & Skibinski, 2004;Bierne et al., 2003;Faure, David, Bonhomme, & Bierne, 2008;Hilbish, Carson, Plante, Weaver, & Gilg, 2002).

Although previous population genetic studies have uncovered clear evidence for hybridization in European mussels, estimates of the magnitude of introgression have tended to be somewhat crude due to the use of a single diagnostic marker (Me15/16, Inoue, Waite, Matsuoka, Odo, & Harayama, 1995) or small panels of diagnostic, partially diagnostic or otherwise informative loci. A related problem is that genetic markers designed to discriminate between species may suffer from ascertainment bias when used to quantify patterns of genetic variability across species (Heslot, Rutkoski, Poland, Jannink, & Sorrells, 2013;Lachance & Tishkoff, 2013). In principle, both of these issues can be circumvented by subjecting the pure species together with any potential hybrids to restriction site‐associated DNA (RAD, Baird et al., 2008) sequencing, an approach for genotyping thousands of essentially random genome‐wide distributed SNPs.

An important aspect of genetic variability that has been linked to fitness variation in many species is heterozygosity (Chapman, Nakagawa, Coltman, Slate, & Sheldon, 2009;David, 1998;Hansson & Westerberg, 2002;Szulkin, Bierne, & David, 2010). Literally hundreds of studies of wild organisms ranging from shellfish to birds and mammals have uncovered heterozygosity fitness correlations (HFCs) for a wealth of traits ranging from early survival through growth to reproductive success (Coltman, Bowen, & Wright, 1998;Pujolar, Maes, Vancoillie, & Volckaert, 2005;Slate, Kruuk, Marshall, Pemberton, & Clutton‐Brock, 2000). In *Mytilus*, over forty such studies have been conducted (reviewed in Koehn, 1991). These consistently point toward positive effects of heterozygosity on energy metabolism and protein synthesis, which in turn influence a multitude of commercially important traits including early growth and feeding rates, viability and reproductive output. However, HFCs remain poorly understood because most studies use too few genetic markers to accurately quantify variation in genome‐wide heterozygosity, or inbreeding (Balloux, Amos, & Coulson, 2004;Kardos, Allendorf, & Luikart, 2014). Fortunately, the large genome‐wide distributed SNP datasets generated by RAD sequencing have proven capable of quantifying inbreeding with far greater precision than small panels of classical genetic markers (Hoffman et al., 2014).

Here, we RAD sequenced mussel populations of unknown ancestry along a European latitudinal cline together with putatively pure reference populations of the three *Mytilus* species in order to characterize genotype frequencies across Europe and to investigate introgression and its effects on genome‐wide heterozygosity. We reconstructed local ancestries with high precision to test a number of hypotheses. First, while previous studies of introgression in mussels focused mainly on the frequency of hybrids in each population (e.g., Bierne et al., 2003;Daguin et al., 2001), our high‐resolution data allowed us to quantify the magnitude of introgression at both the individual and population level. Given that all three *Mytilus* species readily hybridize, we hypothesized that introgression would be widespread, even if the fraction of introgressed alleles might be low in some populations. Second, we evaluated the geographical distribution of commercially damaging *MT* genotypes, which we hypothesized would be more abundant in areas of low salinity. Third, we expected to find a universally positive effect of hybridization on genome‐wide heterozygosity. The overall aims of our study were (a) to inform the mussel industry about the genomic backgrounds of different populations across Europe, which may be important for the selection of potential sources of mussel seed; and (b) to understand the consequences of introgression for genome‐wide heterozygosity, which has previously been linked to variation in commercially relevant traits.

## 2 MATERIALS AND METHODS

### 2.1 Sample collection

**FIGURE 1:**
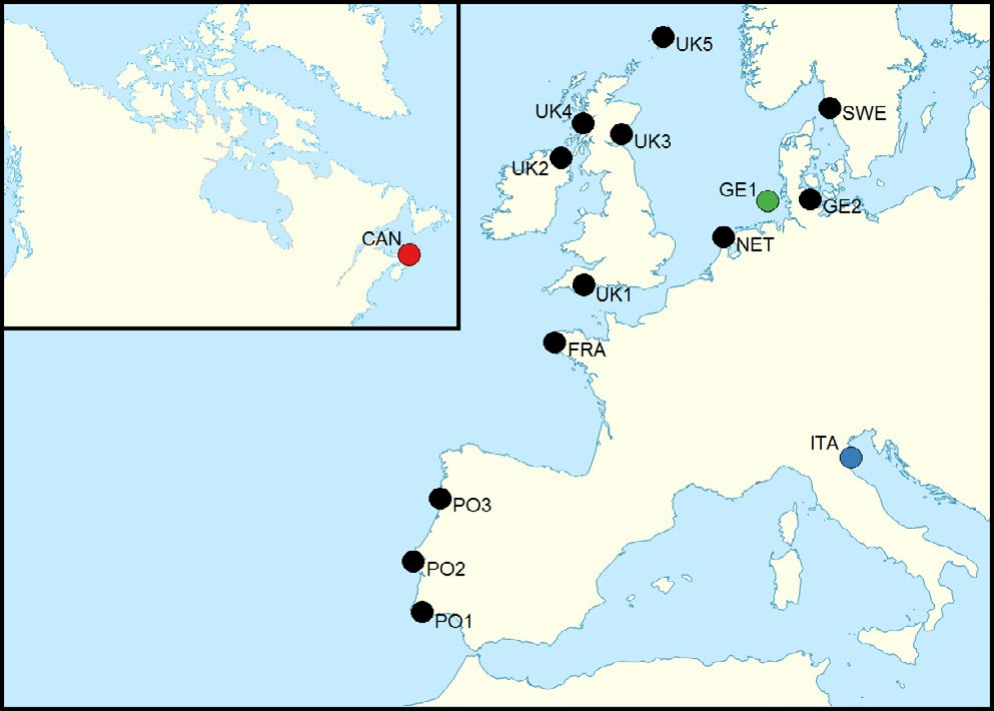
Map showing mussel sampling locations. The black circles represent putatively introgressed populations that were sampled along a European latitudinal cline. The green, blue, and red circles represent putatively pure reference populations of *M. edulis*, *M. galloprovincialis,* and *M. trossulus* (hereafter referred to as *ME*, *MG,* and *MT*) respectively

A total of 262 mussel samples were collected from 13 different sites along the Atlantic coastline of mainland Europe as well as from one site each from the Mediterranean and the Atlantic coast of Canada (Table 1 and Figure 1). These included 12 populations that were previously classified as “potentially introgressed” (Bierne et al., 2003;Daguin et al., 2001) plus three “putatively pure” reference populations of *ME* (GE1), *MG* (ITA), and *MT* (CAN) (Daguin et al., 2001;Stuckas, Stoof, Quesada, & Tiedemann, 2009;Wilson, Matejusova, McIntosh, Carboni, & Bekaert, 2018). All of the samples were of wild origin, with the exception of those from UK5, which originated from a mussel farm.

**TABLE 1:** Information on the *Mytilus* populations that were sampled for this study

Population ID	Sampling location	Population ancestry	Samples passing QC	*H*_o_
ITA	Comacchio, Italy	Pure *MG*	16	0.11
PO1	Faro, Portugal	Potentially introgressed	18	0.1
PO2	Lisbon, Portugal	Potentially introgressed	18	0.1
PO3	Porto, Portugal	Potentially introgressed	18	0.1
FRA	Brest, France	Potentially introgressed	15	0.09
UK1	Exmouth, UK	Potentially introgressed	16	0.09
NET	Texel, Netherlands	Potentially introgressed	17	0.09
GE1	Helgoland, Germany	Pure *ME*	17	0.09
GE2	Kiel, Germany	Potentially introgressed	17	0.09
UK2	Belfast, UK	Potentially introgressed	15	0.09
UK3	St. Andrews, UK	Potentially introgressed	14	0.09
UK4	Oban, UK	Potentially introgressed	18	0.09
SWE	Kristeneberg, Sweden	Potentially introgressed	17	0.09
UK5	Shetland Isl., UK	Potentially introgressed	18	0.09
CAN	Bras d'Or Lake, Canada	Pure *MT*	18	0.13

Note

Population ancestry was assigned a priori as putatively pure *M. edulis* (*ME*), putatively pure *M. galloprovincialis* (*MG*), putatively pure *M. trossulus* (*MT*), or potentially introgressed. The number of samples passing quality checks (QC) and mean observed heterozygosity (*H*_o_) is reported for each population.

### 2.2 DNA extraction, RAD sequencing, and bioinformatic analysis

Whole genomic DNA was extracted from the adductor muscle of each sample using an adapted phenol‐chloroform protocol (Sambrook, Fritsch, & Maniatis, 1989) and shipped to the Beijing genomics institute for RAD sequencing. Libraries were constructed using the restriction enzyme PstI and sequenced on an Illumina HiSeq 4000 to generate a total of 292,239,549 50 bp single‐end reads. After assessing the quality of the demultiplexed sequence reads using FastQC (http://www.bioinformatics.babraham.ac.uk/projects/fastqc/), the reads were de novo assembled using the Stacks 2.2 pipeline (Catchen, Hohenlohe, Bassham, Amores, & Cresko, 2013). Values of the three main parameters –*m*, –*M*, and –*n* were chosen following the optimization procedure described by Rochette and Catchen (2017). Briefly, –*m* was set to three and a range of values for –*M* and –*n* were evaluated. The combination of these parameters for which the number of polymorphic loci present in at least 80% of individuals reached a plateau was defined as optimal. Two different strategies were employed: –*n* was either set as equal to –*M* or one unit greater to account for the possible presence of polymorphisms fixed in one of the three *Mytilus* species (Paris, Stevens, & Catchen, 2017). The optimal combination (*m* = 3, *M* = 5, and *n* = 6) was then selected for analyzing the entire dataset. However, only the 51 samples belonging to the pure populations were used to generate the catalog in order to minimize the potential for noise (Rochette & Catchen, 2017). The raw genotypes were then filtered to retain only biallelic SNPs with genotype quality and depth of coverage greater than five using VCFtools (Danecek et al., 2011), as well as to retain only SNPs genotyped in at least 60% of the individuals. Subsequently, we discarded all SNPs with a depth of coverage greater than twice the mean depth of the raw SNP dataset (>34.4) in order to filter out potentially paralogous loci. Next, all individuals with more than 50% missing data were removed and only variants with MAF greater than 0.01 were retained. Finally, the software PLINK (version 1.9, Purcell et al., 2007) was used to prune out putatively linked loci using an *r* ^2^ threshold of .5.

### 2.3 Genetic analysis of the putatively pure populations

Prior to analyzing the full dataset, we conducted a separate analysis of the putatively pure *Mytilus* samples to validate their use in subsequent introgression analyses. First, we used the R package “ape” (version 5.3, Paradis & Schliep, 2018) to construct a phylogenetic tree based on Euclidean distances followed by hierarchical clustering using Ward's method. Second, we implemented principal component analysis (PCA) using the R package “adegenet” (version 2.1.1, Jombart, 2008;Jombart & Ahmed, 2011). Third, we explored patterns of polymorphism within and among species by quantifying the number of SNPs that were polymorphic within each species and across species. The results of this analysis were visualized in the form of a Venn diagram using the R package “venneuler” (version 1.1, Wilkinson, 2011).

### 2.4 Population genetic structure and introgression

A number of complementary approaches were applied to the full dataset in order to evaluate species composition and introgression. Initially, we investigated the overall pattern of population genetic structure by constructing a phylogenetic tree and by subjecting the full dataset to PCA as described above. We then explored patterns of introgression by conducting a genetic admixture analysis. Specifically, the R package LEA (version 2.6, Frichot & François, 2015) was used to perform sparse non‐negative matrix factorization (sNMF) analysis, which determines the most likely number of genetic clusters (*K*) present in a dataset and outputs individual admixture coefficients (*Q*). sNMF efficiently handles large numbers of markers while in general performing as well as other methods that estimate admixture proportions (Wollstein & Lao, 2015). Five independent runs with an alpha regularization parameter of 100 were performed for each value of *K*, which was set to between one and five, and the best *K* value was determined by calculating cross‐entropy values.

For comparison, we also conducted a formal analysis of introgression using the software ELAI (version 0.99, Guan, 2014). ELAI relies on a hidden Markov model to infer local ancestry for admixed individuals, which is quantified as “ancestry dosage” and has two important advantages over alternative approaches: (a) it does not require phased data and/or mapping distances; and (b) it is capable of dealing with three‐way hybridization scenarios. Five independent replicate runs were implemented for all twelve of the potentially introgressed populations, while the putatively pure *Mytilus* populations were used as “baselines.” This analysis was performed by setting the number of upper clusters (‐C) to three, corresponding to the number of *Mytilus* species present in Europe. The number of lower clusters (‐c) was set to five, as recommended by Guan (2014), the number of admixture generations (‐mg) was set to 40 to account for potential admixture over the last century, and the number of expectation‐maximization (EM) steps (‐s) was set to 40 following the recommendation of Guan (2014). Ancestry dosage values from each of the five runs were then averaged across all individuals within each of the populations to quantify population‐specific fractions of *ME*, *MG* and *MT* ancestry.

Finally, data on salinity and sea surface temperature (SST) were extracted from the E.U. Copernicus Marine Service Information (http://marine.copernicus.eu) and used to test for associations between environmental conditions and the proportions of *ME*, *MG,* and *MT* ancestry across Europe. Specifically, we tested for correlations between each of the three *Mytilus* ancestry values and the two environmental variables while correcting the resulting *p*‐values for the false discovery rate (FDR) as described in Benjamini and Hochberg (1995). We decided to focus on SST because *ME* and *MG* have often been referred to as cold‐temperate and warm‐temperate species respectively, and on salinity because adaptation to low salinity environments has been proposed for *MT* (Riginos & Cunningham, 2005).

### 2.5 Effects of introgression on heterozygosity

To test for differences in genome‐wide heterozygosity among populations with different genetic backgrounds, we used PLINK to calculate observed heterozygosity (*H*_o_) averaged over all loci for each population. We then attributed “main ancestry” to each population depending on whether *ME*, *MG,* or *MT* had the highest mean ancestry dosage value and tested for significant differences in heterozygosity using a *t* test. Subsequently, we investigated the effect of introgression on heterozygosity among populations with different main ancestries. To do so, we constructed a general linear model (GLM) where main ancestry, introgression (measured as the total proportion of ancestry dosage not attributable to the focal species), and their interaction were modeled as predictors of *H*_o_.

Next, we replicated the above analyses at the individual level, this time expressing heterozygosity as standardized multilocus heterozygosity (sMLH), which was calculated using the R package InbreedR (Stoffel et al., 2016). Main ancestry was assigned to each individual according to whether the maximal ancestry dosage was attributable to *ME*, *MG,* or *MT*, and an ANOVA was implemented to test for differences in sMLH among individuals with different genetic backgrounds. We then investigated the effect of introgression on sMLH by constructing a general linear mixed model (GLMM). Here, the response variable was sMLH and individual main ancestry, introgression, and their interaction were fitted as predictors, while sampling location was also included as random effect. The significance of the predictor variables was determined using a parametric bootstrap approach. Specifically, we constructed two alternative models, one including and one excluding the term of interest, and calculated an observed likelihood ratio statistic. We then simulated 1,000 bootstrap replicates based on the null model and used them to generate the null distribution of our test statistic and to calculate a *p*‐value.

## 3 RESULTS

To provide detailed insights into the structure of the *Mytilus* species complex across Europe and to investigate the impact of introgression on genome‐wide heterozygosity, we RAD sequenced a total of 262 mussel samples from fifteen different populations (Table 1). These included twelve populations spanning a European latitudinal cline plus putatively pure reference populations of *ME*, *MG,* and *MT* from Helgoland in Germany, Comacchio in Italy and the Bras d´Or Lake in Canada, respectively. A total of 292,239,549 single‐end 50 bp Illumina sequence reads were generated and de novo assembled into 926,383 RAD loci, which were used to call 1,773,643 raw SNPs. Application of the stringent filtering criteria described in the Materials and methods resulted in a final dataset consisting of 252 samples genotyped at 6,777 SNPs with an average depth of coverage of 12.8.

### 3.1 Species‐level relationships and genetic diversity

**FIGURE 2:**
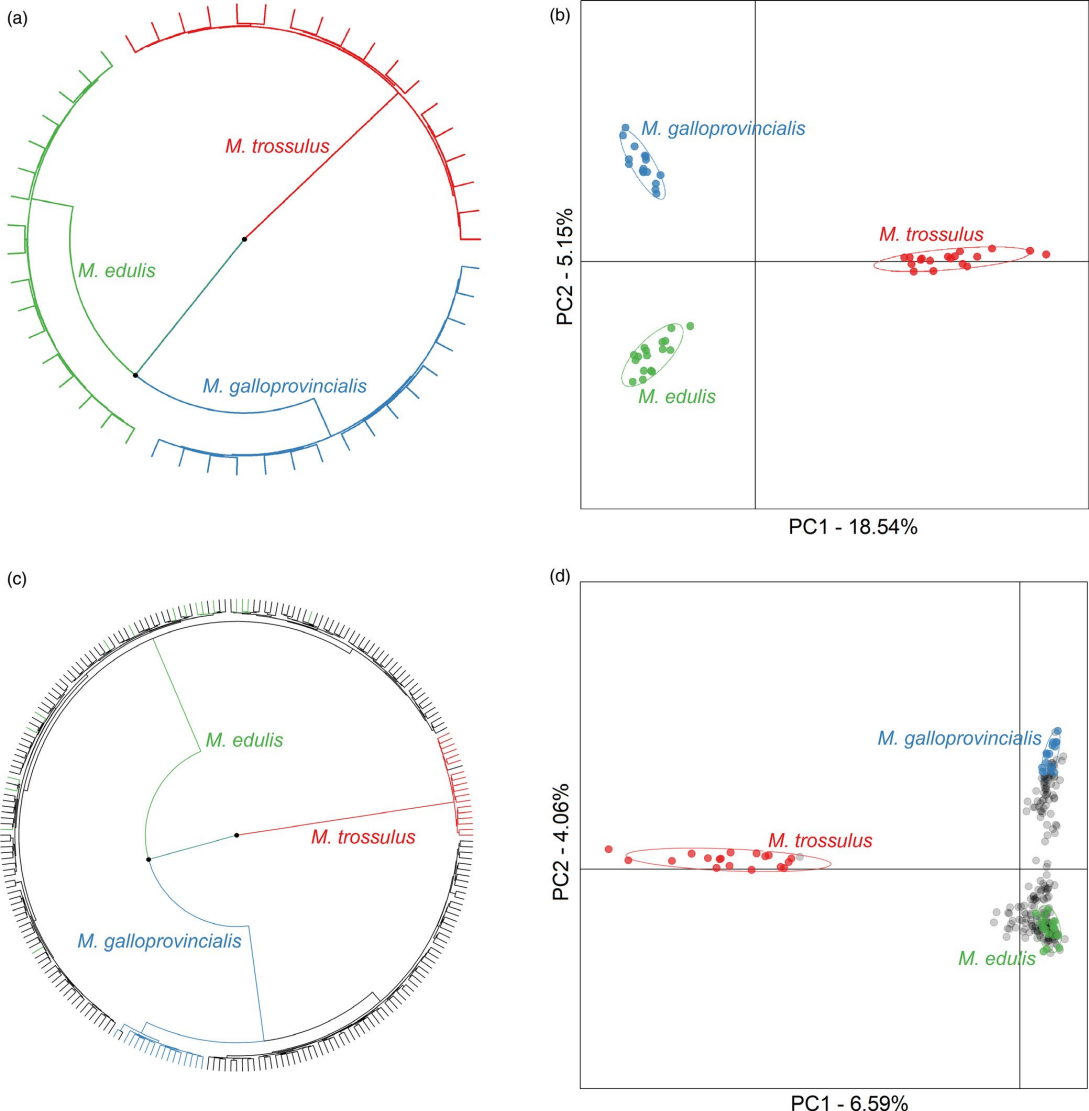
Results of phylogenetic and clustering analyses shown separately for the putatively pure reference populations (panels a and b) and for the full dataset (panels c and d). Panels (a) and (c) show phylogenetic trees, with tree edges representing individuals, color coded according to their ancestry as shown in Figure 1, and nodes with bootstrap support greater than 90% marked by black points. Panels (b) and (d) show scatter plots of individual variation in principal component (PC) scores derived from principal component analysis (PCA). The amounts of variation explained by each PC are given as percentages, and samples are again color coded as shown in Figure 1

In order to verify the species identities of our putatively pure *ME*, *MG*, and *MT* samples, we constructed a phylogenetic tree and subjected the data to principal component analysis (PCA). The RAD dataset clearly resolved all three *Mytilus* species and showed that *ME* is phylogenetically more closely related to *MG* than *MT* (Figure 2a and b). All of the samples grouped as expected based on their putative species identities, suggesting that our species assignments are correct, and none of the samples occupied intermediate positions in the tree, implying an absence of hybrids. Observed heterozygosity was highest for *MT*, intermediate for *MG*, and lowest for *ME* (Table 1). Over half of the loci were polymorphic in a single *Mytilus* species (*n* = 3,544, 52.3%) while around a quarter were polymorphic in two species (*n* = 1,645, 24.3%) and fewer than 10% (*n* = 613) were polymorphic in all three species (Figure S1). 66 loci exhibited fixed differences between *MT* and the other two species and were therefore classified as putatively *MT*‐diagnostic. Further information on these loci including their flanking sequences is provided in Table S1.

### 3.2 Species composition across Europe

Phylogenetic analysis of the full dataset of fifteen populations uncovered three well‐supported clades broadly corresponding to the three *Mytilus* species (Figure 2c). Consistent with the phylogenetic tree of the three pure populations, the *MT* clade (which included the Canadian samples plus a single mussel from the UK) was resolved as an outgroup. The remaining samples formed two clades, corresponding to *ME* and *MG,* respectively. The former did not show any evidence of sub‐structure, while the latter was further divided into two groups comprising the pure *MG* samples and the remaining predominantly southern European samples.

PCA of the full dataset revealed a similar pattern, with the samples clustering into three distinct groups (Figure 2d). Specifically, the first principal component separated the pure *MT* samples from the remaining samples, with the exception of a single British sample, while the second principal component separated *ME* from *MG*. Furthermore, when PC3 was also taken into account, the pure *MG* samples from the Adriatic separated apart from the other populations belonging to the *MG* cluster (Figure S2). Additionally, an appreciable number of individuals occupied intermediate positions between the pure *ME* and *MG* samples, providing a first indication of the presence of introgressed mussels in our dataset.

### 3.3 Patterns of introgression

**FIGURE 3:**
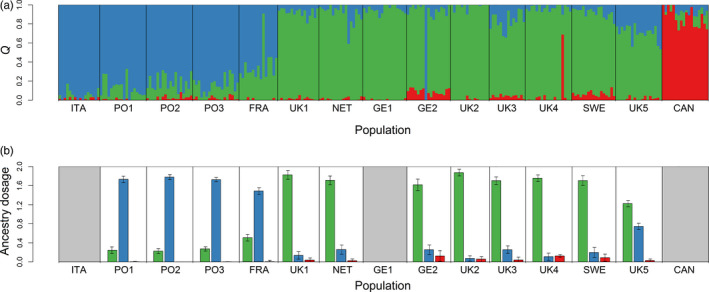
Results of genetic admixture analysis and ancestry inference. Panel (a) shows cluster membership coefficients (*Q*) where each individual is represented by a vertical line partitioned into segments of different color, the length of which indicate the posterior probability of membership in each cluster. Panel (b) shows mean ancestry dosage values for *ME* (green), *MG* (blue), and *MT* (red) for each population. Average values from five independent simulations are plotted together with their standard errors. Data are not shown for ITA, GE1, and CAN as these were used as pure reference populations of *MG*, *ME,* and *MT* respectively

To investigate geographical patterns of introgression, we used sNMF to assign individuals to genetic clusters and to derive admixture coefficients. The most likely number of genetic clusters (*K*) in our dataset was three (Figure S3), corresponding to the three *Mytilus* species. Membership coefficients (*Q*) for the inferred clusters are summarized in Figure 3a, where each vertical bar represents a different individual and the relative proportions of the different colors indicate the probabilities of being assigned to each cluster. Samples from the three Portuguese populations (PO1, PO2 and PO3) and from northern France (FRA) were predominantly assigned to the *MG* cluster, with the remaining ancestry being largely attributable to *ME*. The remaining northern European populations were predominantly assigned to the *ME* cluster, although two populations from the east coast of Scotland and the Shetland islands carried somewhat larger *MG* contributions and a single individual from Kiel in Germany was assigned as pure *MG*.

Small amounts of *MT* ancestry were also detected in several northern European populations (Figure 3a). The fraction of ancestry attributable to *MT* was generally below 0.1, although the two populations closest to the Baltic Sea (GE2 and SWE) had higher *Q* values in the order of 0.1–0.2 and a single individual from Oban (UK4) on the west coast of Scotland had a *Q* value of 0.72. To investigate further, we examined the subset of 66 loci that were found to be diagnostic of *MT* in our previous analysis of the pure populations. We found that mussel populations from Oban, Kiel, and Kristeneberg carried *MT‐*diagnostic alleles at between ten and 25 of these loci (Figure S4), suggesting that the signal of introgression is not an artifact of allele frequency differences at loci that are not actually diagnostic of *MT*. Slightly fewer diagnostic alleles were detected in the Shetland Islands, around the UK, and in the Netherlands, while only 2–3 diagnostic alleles were found in northern France and Portugal, either suggesting that *MT* introgression occurs in a limited way as far south as Faro, or that a small fraction of these loci may not be strictly diagnostic.

Next, we conducted a formal analysis of hybridization by quantifying ancestry dosage values for each individual (Figure 3b). Consistent with the results of the clustering analysis, *MG* accounted for a large proportion of the ancestry of mussels from Portugal and northern France, while *ME* was the dominant species around the coasts of the UK, the Netherlands, Germany and Sweden. Small fractions of *MT* ancestry were also detected in populations from around the coast of the UK and in proximity to the entrance to the Baltic Sea. These patterns are partly explained by environmental variation, as significant associations were found at the population level between SST and the mean ancestry dosage values of *ME* (*r* = −.832, *p* < .01), *MG* (*r* = .84, *p* < .01), and *MT* (*r* = −.778, *p* < .01), while only *MT* introgression showed a significant correlation with salinity (*r* = −.676, *p* < .05).

### 3.4 Effects of introgression on heterozygosity

**FIGURE 4:**
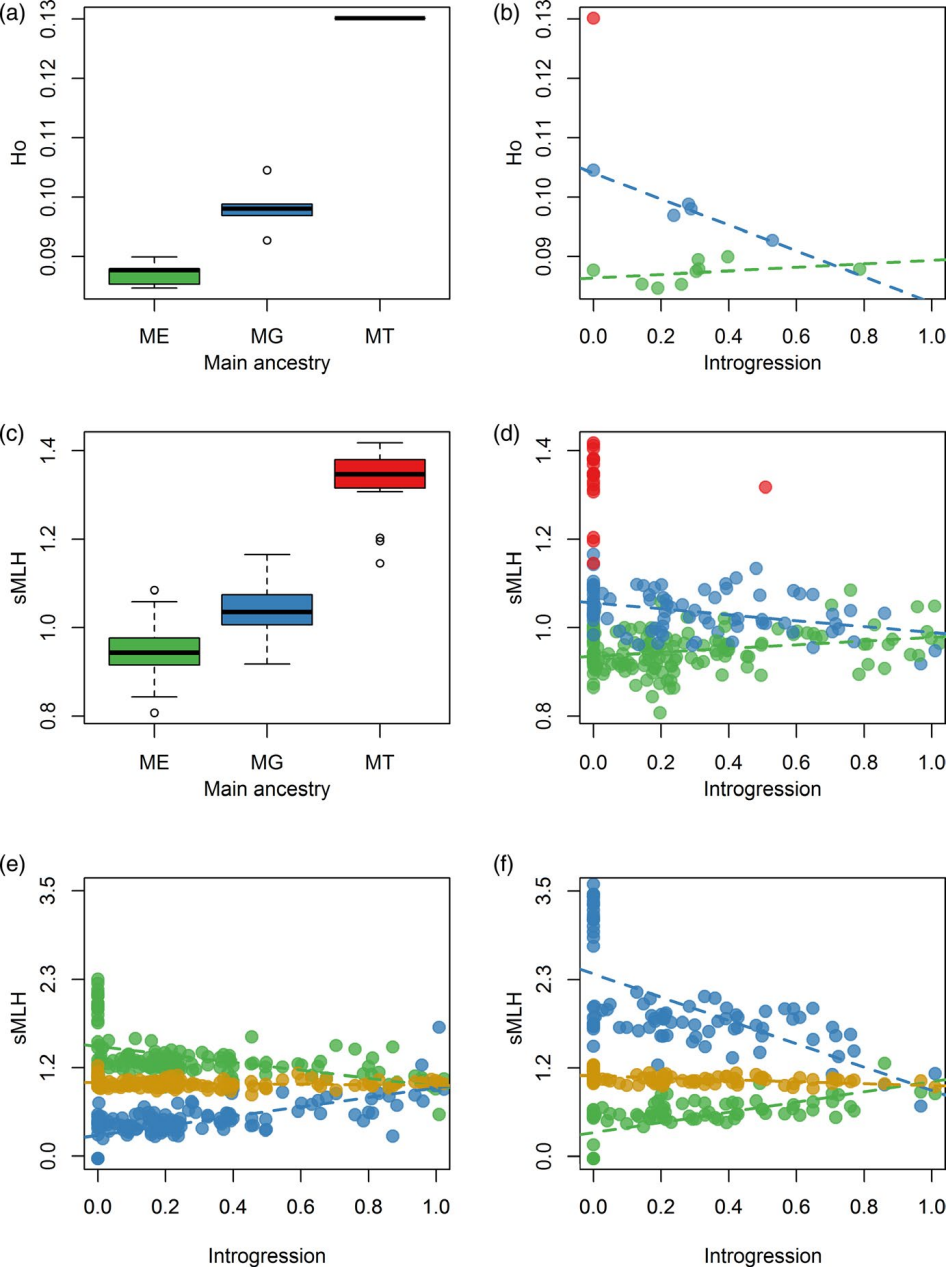
The influence of introgression on genome‐wide heterozygosity. Panels (a) and (b) show the results of population‐level analyses in which heterozygosity was quantified as *H*_o_ (see Materials and methods for details). Panel (a) shows variation in *H*_o_ among populations with different main ancestries. The raw data points are shown together with Tukey box plots (centre line = median, bounds of box = 25th and 75th percentiles, upper and lower whiskers = largest and smallest value but no further than 1.5 * inter‐quartile range from the hinge). Panel (b) shows the relationship between *H*_o_ and the magnitude of introgression, defined as the total proportion of ancestry dosage attributable to the other two *Mytilus* species. Each point represents a population, color coded according to whether its main ancestry was *ME* (green), *MG* (blue), or *MT* (red). The regression lines show the fit of generalized linear models constructed separately for each species. Panels (c) and (d) show the results of individual‐level analyses in which heterozygosity was quantified as standardized multilocus heterozygosity (sMLH). Panel (c) shows variation in sMLH among individuals with different main ancestries, while panel (d) shows the relationship between sMLH and the magnitude of introgression, defined as above. Panels (e) and (f) show how individual sMLH varies with different levels of introgression in mussels whose main ancestry was (e) *ME* and (f) *MG*. In both of these panels, sMLH was calculated separately for loci that were only polymorphic in pure *ME* individuals (“*ME*–SNPs,” shown in green), for loci that were only polymorphic in pure *MG* individuals (“*MG*–SNPs,” shown in blue) and for loci that were polymorphic in both pure *ME* and pure *MG* individuals (“*ME*/*MG*–SNPs,” shown in gold). The regression lines show the fit of generalized linear models constructed separately for each subset of loci

In order to investigate the influence of introgression on genome‐wide heterozygosity, we first assigned "main ancestry" to each population based on whether *ME*, *MG,* or *MT* had the highest mean ancestry dosage values. We then tested for differences in observed heterozygosity (*H*_o_) among populations with different main ancestries. Figure 4a shows that populations dominated by *ME* ancestry had significantly lower *H*_o_ than populations whose main ancestry was *MG* (unpaired *t* test, *t =* −5.45*, p* < .01). The pure *MT* population had the highest overall *H*_o_ although we did not test for statistical significance given that none of the other populations had majority *MT* contributions. For each population, we then quantified the magnitude of introgression as the total proportion of ancestry dosage attributable to the other two *Mytilus* species. Finally, we constructed a GLM of *H*_o_ with main ancestry, introgression and their interaction fitted as predictor variables. All three were highly significant (main ancestry: *F = *139.28*, p* < .01; introgression: *F = *7.36*, p* < .05; interaction: *F = *21.7*, p* < .01), confirming species‐level differences and suggesting that the influence of introgression on *H*_o_ depended on the primary genomic background. Specifically, the introgression of *ME* alleles into populations whose main ancestry was *MG* had a highly significant negative influence on *H*_o_ (*b* = −0.02, *t =* −6.36*, p* < .01) whereas the introgression of *MG* alleles into populations whose main ancestry was *ME* had a weakly positive but nonsignificant effect (*b* = 0.003, *t =* −0.977*, p* = .36, Figure 4b).

Next, we repeated the analysis at the individual rather than the population level. Each individual was assigned main ancestry according to the maximal ancestry dosage attributable to *ME*, *MG*, or *MT*, and individual genome‐wide heterozygosity was quantified as standardized multilocus heterozygosity (sMLH). Again, we found clear differences in heterozygosity among individuals with different main ancestries, with mean sMLH being lowest for individuals whose main ancestry was *ME*, intermediate for individuals whose main ancestry was *MG,* and highest for individuals whose main ancestry was *MT* (ANOVA: *F* = 544.33, *p* < .01, Figure 4c). To investigate the interplay between introgression and heterozygosity at the individual level, we constructed a GLMM where sMLH was the response variable, individual main ancestry, introgression, and their interaction were fitted as predictor variables, and sampling location was included as a random effect. Again, all three predictors were statistically significant (main ancestry, *p* < .01; introgression, *p* = .018; interaction, *p* < .01) with *ME* introgression into individuals whose main ancestry was *MG* having a negative influence on sMLH (*b* = −0.05, *p* < .01), whereas *MG* introgression into individuals whose main ancestry was *ME* had a positive influence on sMLH (*b* = 0.04, *p* < .01, Figure 4d).

To understand why introgression does not increase genome‐wide heterozygosity in both species, we conducted a more detailed analysis focusing on *ME* and *MG*. For this, we exploited information from the pure populations to select three mutually exclusive subsets of SNPs. The first comprised loci that were only polymorphic in pure *ME* individuals (hereafter termed “*ME*–SNPs,” *n* = 1,592). The second comprised loci that were polymorphic in both pure *ME* and pure *MG* individuals (hereafter termed “*ME*/*MG*–SNPs,” *n* = 1,415). The third comprised loci that were only polymorphic in pure *MG* individuals (hereafter termed “*MG*–SNPs,” *n* = 1,501). We then calculated sMLH separately for each class of SNP and investigated how the resulting values were influenced by introgression separately for each species. For individuals whose main ancestry was *ME*, the decrease of sMLH at *ME*–SNPs with increasing introgression was less pronounced than the increase of sMLH at *MG*–SNPs (*ME*–SNPs: *b* = −0.51, *p* < .01; *ME*/*MG*–SNPs: *b* = −0.03, *p* = .047; *MG*–SNPs: *b* = 0.61, *p* < .01; Figure 4e). By contrast, for individuals whose main ancestry was *MG*, the decrease of sMLH at *MG*–SNPs with increasing introgression was more pronounced than the increase of sMLH at *ME*–SNPs (*ME*–SNPs: *b* = 0.66, *p* < .01; *ME*/*MG*–SNPs: *b* = −0.12, *p* < .01; *MG*–SNPs: *b* = −1.51, *p* < .01; Figure 4f). This suggests that the balance of the contributions of *ME*–SNP and *MG*–SNP heterozygosity toward genome‐wide heterozygosity may shift depending on the main genetic background and the magnitude of introgression.

## 4 DISCUSSION

We used RAD sequencing to obtain detailed insights into the genetic composition of the *Mytilus* species complex in Europe. We found evidence for widespread introgression, particularly between *ME* and *MG*, although small contributions of *MT* ancestry were also detected across much of northern Europe. Moreover, introgression had opposing effects on genome‐wide heterozygosity depending on the primary genetic background. As *MT* is considered a commercially damaging species (Scott et al., 2010;Scottish Government, 2014) and heterozygosity is known to impact commercially important traits in *Mytilus* (Koehn, 1991), our findings may have implications for mussel aquaculture.

### 4.1 Reference populations

Many of our analyses relied upon inferences derived from pure reference populations of *ME*, *MG*, and *MT*. We therefore carefully selected reference populations that had been described as pure in the literature (Daguin et al., 2001;Stuckas et al., 2009;Wilson et al., 2018). We specifically chose a Canadian *MT* reference population as opposed to a Baltic one because *MT* and *ME* have extensively mixed in the Baltic, leading to complete replacement of mitochondrial genomes and a hybrid swarm structure (Kijewski et al., 2006;Väinölä & Strelkov, 2011). To confirm the validity of our reference samples, we conducted a phylogenetic analysis, which resolved *ME* and *MG* as sister groups and *MT* as an outgroup. This pattern is consistent with previous molecular and morphological studies (Barsotti & Meluzzi, 1968;Heath, Rawson, & Hilbish, 1995;Vermeij, 1991). Furthermore, the pattern of grouping of individual samples suggested they had all been correctly assigned to species. This is important because a similar study clustered one out of five pure *MT* reference individuals from Penn Cove in the USA together with pure *ME* individuals from Scotland (Wilson et al., 2018), implying that it may be relatively easy to incorrectly assign individual mussels to species based solely on their provenance.

### 4.2 Species distributions and introgression

In line with previous studies based on smaller numbers of mitochondrial or nuclear genetic markers, clear species partitioning was found between southern and northern Europe. Specifically, *MG* ancestry predominated in the Mediterranean, along the coast of the Iberian Peninsula and in Brittany, consistent with Faure et al. (2008), Bierne et al. (2003) and Sanjuan et al. (1994), whereas *ME* was the dominant species across much of northern Europe, as previously shown by Zbawicka et al. (2012) and Daguin et al. (2001). Although we did not find any pure *MT* individuals in our dataset, small fractions of *MT* ancestry were apparent across much of northern Europe and in particular around the entrance to the Baltic as well as in northern Scotland. Prevailing environmental conditions are likely to play a role in explaining these distributions, as ancestry dosage was associated with SST in all three species as well as with salinity in *MT*, consistent with previous work by Riginos and Cunningham (2005). However, the transport of spat in ocean currents or via shipping may also contribute toward the local composition of mussel populations (Stuckas et al., 2017), as appears to be the case in Svalbard where mussels carry large amounts of *MG* ancestry despite *ME* dominating the surrounding areas (Mathiesen et al., 2017).

Among the southern, predominantly *MG* populations, we found a general tendency for *ME* introgression to increase with increasing latitude. However, this pattern may be an artifact of our sampling design, as our dataset does not have the spatial resolution to capture complexities that are known to be present in this system. For example, instead of a single transition occurring from *MG* to *ME* along the western Atlantic seaboard, a mosaic hybrid zone is present that comprises three separate transitions (Bierne et al., 2003;Faure et al., 2008). Our dataset was unable to capture this fine‐scale heterogeneity, although we did find that *MG* ancestry was relatively high in Brittany, consistent with this part of northwestern France constituting an “*MG* island” surrounded by predominantly *ME* populations (Bierne et al., 2003;Faure et al., 2008).

Among the northern, predominantly *ME* populations, considerable geographical variation was found in the magnitude of *MG* introgression. This is again consistent with previous studies documenting a mosaic structure across the UK (Gardner & Skibinski, 1988;Gosling & McGrath, 1990;Hilbish et al., 2002;Skibinski, Beardmore, & Cross, 1983;Wilhelm & Hilbish, 1998). Given that *MG* is considered a warm‐temperate species (Michalek et al., 2016), we were initially surprised to find relatively large amounts of *MG* ancestry in two of the most northerly UK populations, St. Andrews and the Shetland Islands. However, high frequencies of *MG* alleles have been documented at even higher latitudes, possibly due to oceanographic features or human‐mediated transport (Mathiesen et al., 2017). A role of humans also cannot be discounted in northern Scotland as our sample from the Shetland Islands originated from a farmed population that has been augmented with spat from other localities (Michael Tait, personal communication).

We also captured a pervasive but low‐level signal of *MT* ancestry across most of northern Europe. Previous studies have shown that *MT* alleles occur at high frequency in the Baltic (Kijewski et al., 2006, 2019;Väinölä & Strelkov, 2011;Zbawicka et al., 2012;Zbawicka et al., 2014) as well as in some parts of Norway and Greenland (Mathiesen et al., 2017;Wenne et al., 2016). Additionally, *MT* introgression was implicated in the recent collapse of the mussel farming industry at Loch Etive in western Scotland (Beaumont et al., 2008) and has since been documented at several Scottish locations including Highland and Argyll (Beaumont et al., 2008;Michalek et al., 2016). However, our data are suggestive of small contributions of *MT* ancestry not only around the Baltic and the Scottish coasts, but also in the Netherlands, southwest England, Northern Ireland, and the Shetlands. In addition, a single mussel from Oban (out of a total of 18 individuals from this location) had almost 75% *MT* ancestry. Overall, our results imply that *MT* alleles may be more widespread than was previously appreciated.

### 4.3 Introgression and heterozygosity

Previous studies of the *Mytilus* complex in Europe have often neglected genetic diversity, partly due to the unsuitability of diagnostic markers for quantifying genome‐wide patterns, but also due to the risk of ascertainment bias when markers developed in one species are applied to another (Heslot et al., 2013;Lachance & Tishkoff, 2013). We avoided these issues by simultaneously de novo assembling RAD sequencing data from all three species and their putative hybrids. This approach should produce relatively unbiased estimates of genome‐wide variation and thus allow comparative analysis of populations with varying ancestries. As a wealth of previous studies have linked heterozygosity to variation in commercially relevant traits in mussels (reviewed by Koehn, 1991), we focused specifically on the influence of introgression on genome‐wide heterozygosity, which under most circumstances can be reliably inferred from several thousand unlinked SNPs (Hoffman et al., 2014;Kardos, Taylor, Ellegren, Luikart, & Allendorf, 2016).

Highly concordant results were obtained regardless of whether the data were analyzed at the level of populations or individuals, with *ME* having the lowest heterozygosity, *MG* having intermediate heterozygosity, and *MT* having the highest heterozygosity. This is in line with Gardner (1994) who found that allozyme heterozygosity was higher in *MG* than *ME*, as well as with Zbawicka et al. (2014) who reported higher levels of SNP heterozygosity in *MT* relative to *ME*. However, our results are at odds with two other studies documenting comparatively low levels of heterozygosity in *MT* (Mathiesen et al., 2017;Zbawicka et al., 2012). One possible explanation for this discrepancy could be ascertainment bias, as Mathiesen et al. (2017) used SNPs that were mainly discovered in *ME*. Similarly, the majority of SNPs analyzed by Zbawicka et al. (2012) were fixed for a single allele in a pure *MT* population and it is therefore unclear to what extent these loci are representative of the genetic variability of *MT*. Our study should be relatively unaffected by issues relating to preascertained markers, both because our pools of pure individuals were equally large and because RAD sequencing allows thousands of SNPs to be genotyped regardless of the main genetic background or degree of introgression.

One potential drawback of our approach, however, was that the flanking sequences of our RAD loci were too short (approx. 45bp) to allow reliable mapping to a reference genome. Consequently, our SNPs are not accompanied by positional information and functional annotations are lacking for any SNPs that may reside in genes. We do not see this as a major drawback of our study as we were primarily interested in genome‐wide patterns as opposed to the role of specific genomic regions. Nevertheless, more detailed studies of the genomic landscape of introgression are essential for improving our understanding of adaptive phenotypic variation and selection in *Mytilus*.

Somewhat counterintuitively, introgression appears to have contrasting effects on genome‐wide heterozygosity depending on the *Mytilus* species in question. In populations or individuals whose main ancestry was *ME*, we found that the introgression of *MG* alleles was associated with an increase in heterozygosity. By contrast, the introgression of *ME* alleles into populations or individuals whose main ancestry was *MG* was associated with a decrease in heterozygosity. This pattern appears to be a reflection of species‐level differences in heterozygosity and of the balance between the increase of heterozygosity caused by the introgression of new alleles versus the loss of heterozygosity as the primary genetic background is progressively diluted.

### 4.4 Implications for mussel aquaculture

In Europe, seed supply for mussel production relies either on natural local recruitment or on the transfer of spat from shellfish farms (Michalek et al., 2016;Śmietanka et al., 2004). Molecular genomic tools such as RAD sequencing could therefore assist the aquaculture industry by providing information in support of decisions such as where to locate mussel farms and where to source mussel spat. Our study suggests that RAD sequencing is capable of providing detailed information on stock structure and introgression in *Mytilus*. Although we focused primarily on wild mussel populations, it is not difficult to envisage how reduced representation sequencing or related approaches might be applied in an industrial setting, for example to characterize the genetic composition of commercial mussel stocks, to assist in the selection of genetic material for cultivation, or to improve our understanding of how commercially important traits vary among mussels with different genomic backgrounds.

Particularly undesirable for cultivation are mussels carrying appreciable fractions of *MT* ancestry (Scott et al., 2010;Scottish Government, 2014) due to their poor quality meat and fragile shells (Beaumont et al., 2008). Consequently, information on the geographic distribution and magnitude of introgression of *MT* alleles will be of interest to the mussel industry. RAD sequencing allowed us to detect small amounts of *MT* ancestry in several northern European populations, including localities where the presence of *MT* had not previously been reported. Data such as these may contribute toward efforts to minimize the spread of *MT* by helping mussel producers to make more informed decisions about where to source their spat. Our findings also highlight the need for further screening for the presence of *MT* genotypes, particularly around northern European coastlines.

Finally, we uncovered evidence for widespread introgression between *ME* and *MG* and could show that introgression between these species can have rather complex effects on overall levels of genome‐wide heterozygosity. While the commercial implications of these findings may not be immediately obvious, it is important to recognize that the primary genetic background (Bierne, Bonhomme, Boudry, Szulkin, & David, 2006;Coustau, Renaud, Maillard, Pasteur, & Delay, 1991;Gardner, 1994;Hilbish, Bayne, & Day, 1994;Skibinski, 1983), hybridization (Bierne et al., 2006;Gardner, 1994;Gardner, Skibinski, & Bajdik, 1993), and heterozygosity (Koehn, 1991) all have substantial effects on fitness traits such as growth rate, viability, and productivity in European mussels. Furthermore, although heterozygosity tends to be positively associated with fitness within species, the increase in heterozygosity that occurs when two species interbreed can result in a variety of outcomes ranging from hybrid vigor to outbreeding depression (Chapman et al., 2009). Even in *Mytilus* where hybridization has been extensively investigated, contrasting fitness outcomes have been described, with one study finding that introgression between *ME* and *MG* increased fitness (Gardner, 1994), another documenting intermediate fitness in F1 hybrids relative to the two parental species (Gardner et al., 1993), and a third study reporting high levels of larval mortality in F2 hybrids (Bierne et al., 2006). Given the degree of admixture observed in many of the sampled populations in the current study, we believe that a strong case could be made for further studies of the phenotypic effects of introgression in the *Mytilus* complex. RAD sequencing would offer an alternative to using artificial crosses by allowing mussels with different proportions of *ME*, *MG*, and *MT* ancestry (selected on the basis of their ancestry dosage values) to be raised in a common‐garden setup.

## CONFLICT OF INTEREST

None declared.

## DATA ACCESSIBILTY

The raw sequence reads used to generate the results of this study are available at the Short Read Archive (SRA accession: PRJNA615219).

## Supporting information

Fig S1

Fig S2

Fig S3

Fig S4

Table S1
