# Supplementary figures and images for: Genome‐wide insights into introgression and its consequences for genome‐wide heterozygosity in the Mytilus species complex across Europe
Source: Evol Appl. 2020 Apr 24;13(8):2130–42. doi: 10.1111/eva.12974 (PMC7463347; doi:10.1111/eva.12974)

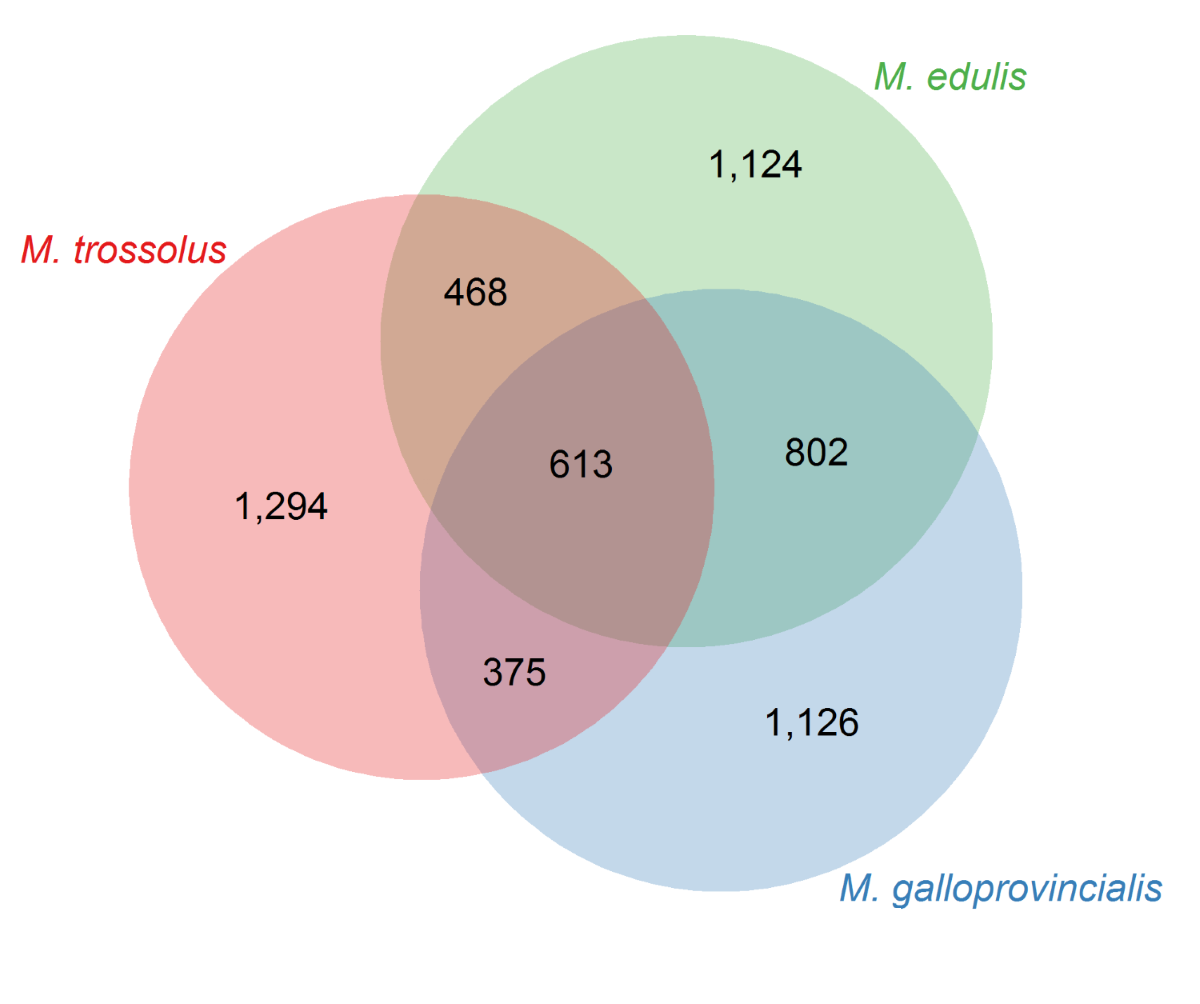

Supplement: Supplementary file 1 — Fig S1 [file EVA-13-2130-s001.tiff]

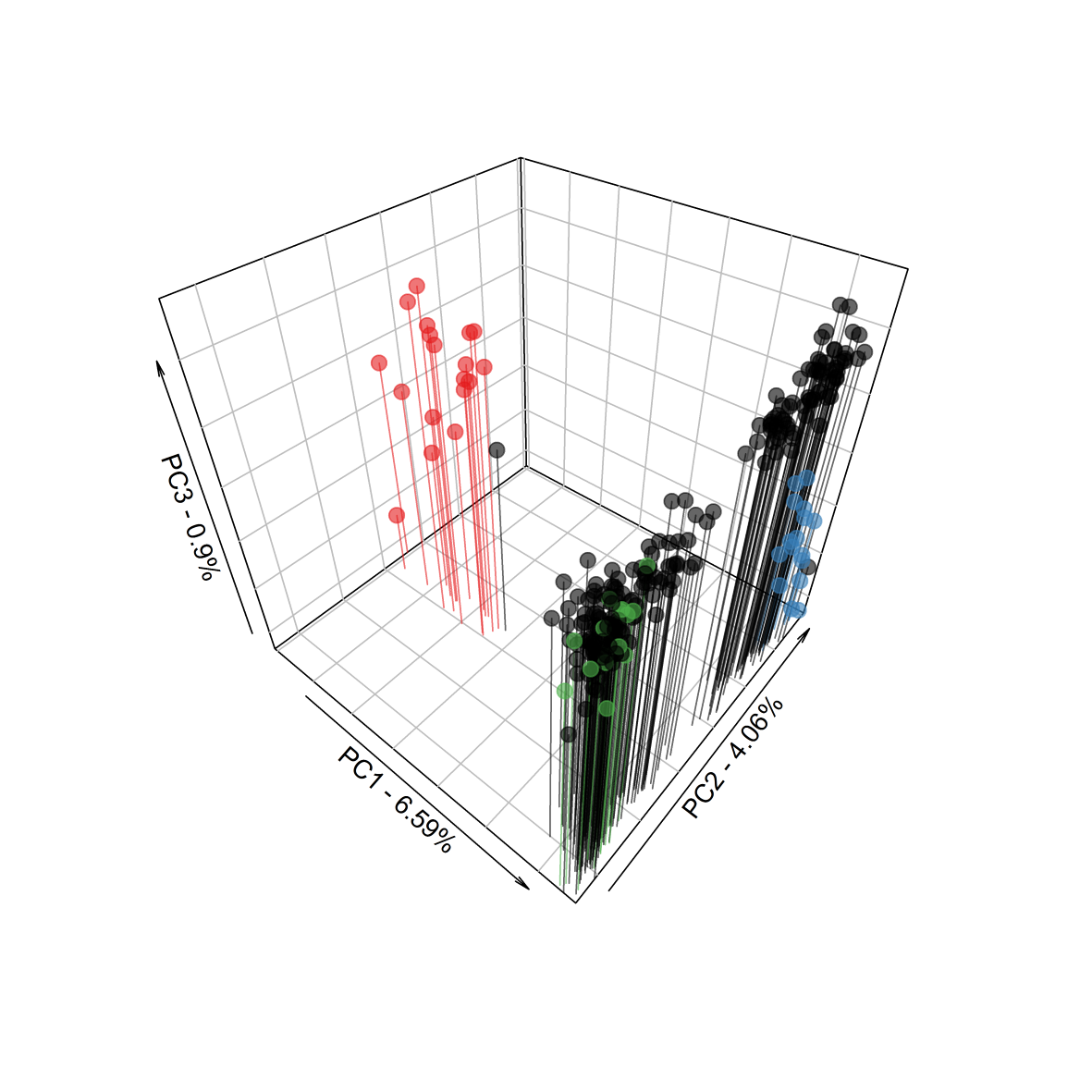

Supplement: Supplementary file 2 — Fig S2 [file EVA-13-2130-s002.tiff]

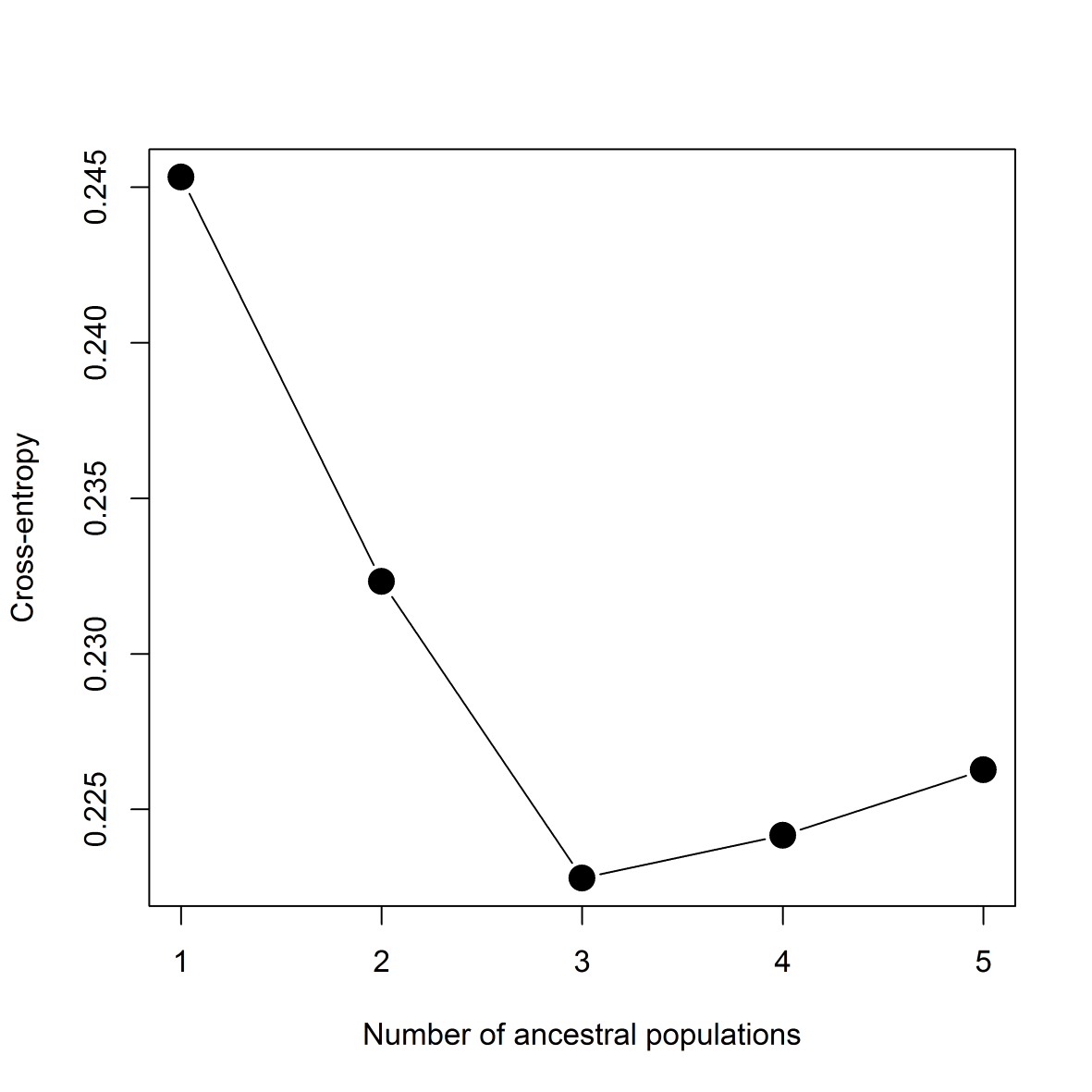

Supplement: Supplementary file 3 — Fig S3 [file EVA-13-2130-s003.tiff]

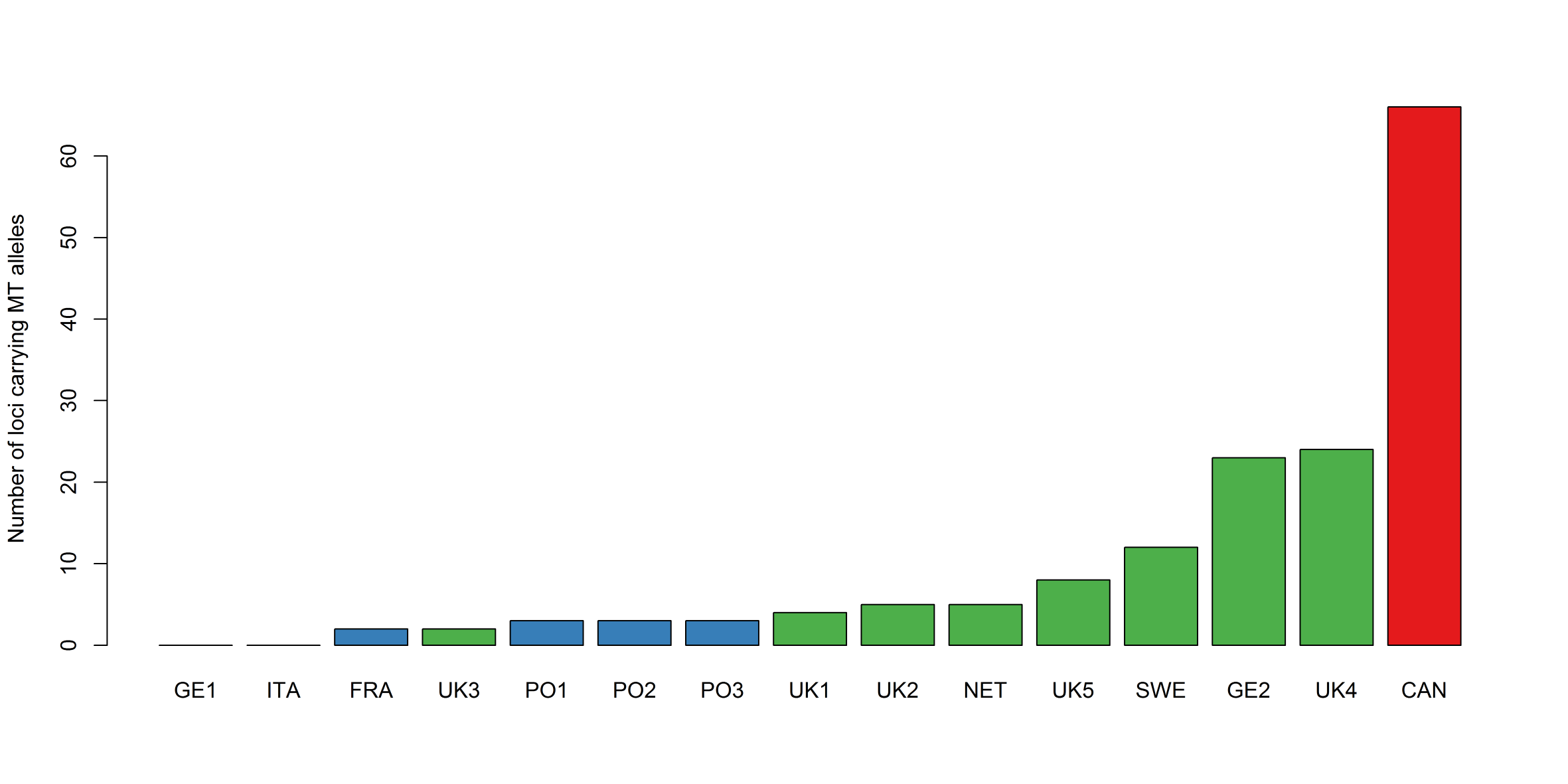

Supplement: Supplementary file 4 — Fig S4 [file EVA-13-2130-s004.tiff]
